# Biological effects and mechanisms of shortwave radiation: a review

**DOI:** 10.1186/s40779-017-0133-6

**Published:** 2017-07-20

**Authors:** Chao Yu, Rui-Yun Peng

**Affiliations:** Beijing Institute of Radiation Medicine, Beijing, 100850 China

**Keywords:** Shortwave, Electromagnetic radiation, Biological effects

## Abstract

With the increasing knowledge of shortwave radiation, it is widely used in wireless communications, radar observations, industrial manufacturing, and medical treatments. Despite of the benefits from shortwave, these wide applications expose humans to the risk of shortwave electromagnetic radiation, which is alleged to cause potential damage to biological systems. This review focused on the exposure to shortwave electromagnetic radiation, considering in vitro, in vivo and epidemiological results that have provided insight into the biological effects and mechanisms of shortwave. Additionally, some protective measures and suggestions are discussed here in the hope of obtaining more benefits from shortwave with fewer health risks.

## Background

Shortwave (SW) is a part of the electromagnetic spectrum, and it has a frequency range varying from 3 MHz to 30 MHz, which belongs to the high frequency (HF) band. This HF band is frequently used in military radars, radio transmissions and industrial equipment. Additionally, several technologies based on the delivery of SW radio frequency energy are available for therapeutic medical applications [1–3].

However, it cannot be ignored that the non-ionizing radiation of SW with HF may damage biological tissues by non-thermal or thermal mechanisms [4–6]. It is known that a rapidly moving electromagnetic field (EMF) can be a health hazard when the energy level is sufficiently high enough, but the variety of electromagnetic devices with different radiation intensities makes the biological effects inconsistent. Although numerous studies about the adverse effects of electromagnetic radiation have been reported in the past decades, the biological effects of radiation remain controversial. Given the potential risk of electromagnetic radiation, many countries have determined the safe levels of exposure for the general public and occupational workers [7, 8].

Here, we focused on the exposure to SW radiation and reviewed the present studies; we aimed to provide a comprehensive summary of the biological effects of SW.

## SW-EMF and medical treatment

The development of bioelectromagnetics has facilitated the application of various EMFs on medical treatment, and SW-EMF is broadly available. Many studies, including basic science and clinical evidence, have reported the beneficial effects of SW-EMF on cancer treatment [1, 9–14], wound repair [2, 15] and pain control [3, 16].

### Therapeutic effect on cancer

Cancer treatment is always a challenge, and scientists have been exploring new therapeutic methods, including SW-EMF treatment. Several studies have demonstrated that SW-EMF facilitated the treatment of some cancer types. Jimenez et al. [9] exposed mice carrying subcutaneous hepatocellular carcinoma (HCC) xenografts to 27.12 MHz of EMF modulated at specific frequencies, 3 h daily for 6 weeks. The specific absorption rate (SAR) of SW-EMF was 0.4 W/kg. The results showed that in vivo HCC was significantly inhibited in mice exposed to modulated SW-EMF. Additionally, the modulated HCC-specific and breast cancer-specific frequencies of SW-EMF significantly decreased the proliferation of HCC and breast cancer cells, but the non-malignant cell lines of the above two tumors were not affected by the same frequencies [10]. Another clinical experiment study concluded that treatment with intrabuccally administered amplitude-modulated SW-EMF was effective for palliating symptoms and prolonging the survival period of patients with advanced HCC [1]. From these studies, we concluded that some cancer types had special SW response frequencies, while others tended to be unresponsive at any frequency. This difference may stem from their different proliferation properties or cancer cell phenotypes [12]. These above studies all focused on the non-thermal therapeutic effects of SW. Additionally, some reports have demonstrated that the selective hyperthermia induced by SW was also effective in cancer treatment [13, 14]. Curley et al. [14] reported that non-invasive SW treatment at 13.56 MHz with 1–20 KeV/m^2^ field inhibited tumor progression in animals and enhanced the anticancer effect of chemotherapy.

### Promoting effects on wound repair

Many studies have reported that non-thermal EMF could promote wound healing. The effect of pulse modulated SW-EMF on skin wound repair was evaluated in a full-thickness skin wound model in rats, and the tensile strength of new growing skin was tested [15]. After the exposure to a 65-μs burst of 27.12 MHz sinusoidal waves inducing a 1-G peak magnetic field for 30 min twice daily for 21 days, the mean tensile strength of the treated group was 48% (*P* < 0.01), which was greater than that of the control. The finding successfully demonstrated that the stimulation of SW-EMF helped reconstruct and restore wound skin. Additionally, SW-EMF had an inhibition effect on the release of inflammatory cytokines, suggesting that an alleviative inflammatory response and rapid recovery could be expected [2, 3, 16, 17]. In a double-blinded, placebo-controlled, randomized study, 32 postoperative patients were treated with a pulse EMF device and sham device [2]. After 1 h of exposure to the 27.12 MHz pulse EMF with repetition at 2 bursts/s and 5 V/m electric field, the interleukin (IL)-1α and IL-1β in wound exudates of the sham group were approximately 2.2-fold higher than the EMF group, which indicated a reduced inflammatory response in the EMF group.

### Postoperative pain management

The management of postoperative pain is indispensable, and the attempt of the physical method, including the EMF therapy to ease the pain, is another choice. In a clinical study, a pulse-modulated SW-EMF was applied in human subjects after breast reduction surgery [18]. The EMF device had settings of 27.12 MHz, 1000 pulses/s and a 100 μs burst width, and the peak burst power was 9.8 mW covering a 100 cm^2^ area. After 20 min of treatment every 4 h, the pain scores of the visual analog scale on postoperative days 1 and 3 in the EMF group were significantly lower than that of the placebo group. In addition, an early study reported the pulsed EMF therapy for persistent neck pain (greater than 8 weeks) [19]. Low-power pulsed SW (approximately 27 MHz and 1.5 mW/cm^2^) was chosen for a safe 8 h/d treatment. After 3 weeks, the pain scale (*P* < 0.023) and range of movement (*P* < 0.002) improved significantly in the EMF group compared to the controls. The effect of SW-EMF on pain relief may be related to the reduced inflammatory response and improved microcirculation [3, 20]. If these findings could be explored further, the pain management with EMF would reduce the dose and side effects of pain medication.

## SW radiation and health hazards

Although some types of low-power EMF were demonstrated to be of benefit to medical treatments, it was likely that electromagnetic radiation with a sufficiently high level of energy could be harmful to human health, and there were no clear dividing lines between positive and detrimental radio frequency energy. Numerous studies and epidemiological investigations have focused on the potential health hazards of SW radiation.

### Central nervous system

The central nervous system was thought to be one of the systems that is sensitive to electromagnetic radiation, and the impairment was mainly characterized by brain dysfunction [4, 21–25]. An epidemiological study tested the electroencephalogram (EEG) characteristics in 98 workers exposed to high frequency (3–30 MHz) EMF for more than 2 years [23]. Compared with 60 healthy adults without any occupational exposure, the EEG showed that the numbers of slow-wave (θ and δ waves) and abnormalities among exposed workers were significantly higher. Additionally, exposed workers had a higher incidence of nervous system symptoms (i.e., headache, dizziness, insomnia). Another study reported the effect of a broadcast transmitter with SW-EMF (6–22 MHz) on the sleep quality in a nearby human population sample [24]. Fifty-four volunteers were followed for 7 days each before and after shut-down of the broadcast transmitter. The self-rated sleep quality was evaluated daily using a visual analog scale. It showed an improved sleep quality, especially in poor sleepers, when the transmitter was shut down. Additionally, an animal experiment investigated the neurobehavioral impact of SW-EMF (30 MHz, 20 mW) on mice [25]. The mice were exposed to weak EMF for 8 h and their behaviors were observed and recorded. The results showed that their rest period was markedly reduced from 36.09 ± 3.48 s/min to 32.44 ± 4.89 s/min (*P* < 0.01). The reasons for these brain dysfunctions induced by SW radiation remained unclear, but they may involve abnormal synthesis and release of neurotransmitters as well as disordered electrical activity in nerve cells.

### Cardiovascular system

The effect of EMF on the cardiovascular system has been a subject of interest because of the electric impulse properties of electrocardio conduction, which allegedly could have interference by exogenous electromagnetic pulses of certain energy [26–28]. An epidemiological investigation presented the results of electrocardiogram (ECG) changes of 138 radiomen who had worked in EMF (134–510 MHz, average electrical field intensity was 18.5 V/m) for an average of 14 years [29]. Compared with 72 workers in the control group, the result showed that radiomen had a significantly higher incidence of abnormal ECG, particularly sinus arrhythmia (*P* < 0.01). Ke et al. [30] also found that the male crews who had worked in the electromagnetic radiation environment (electrical field intensity was 2.73–20.50 V/m) for an average of 4.4 years had a significantly lower heart rate compared to controls. There were still some negative results on the effects of SW radiation on ECG. Chen et al. [31] researched the effect of 27.2 MHz of SW radiation on ECG in female workers at a shoe factory. Two hundred and 25 workers exposed to a high frequency field (the 6-min electric field intensity was approximately 64 V/m) were chosen as the exposure group. Compared with control group of 100 workers without exposure from the same factory, they did not find any significant difference in the ECG changes when several known risk factors for heart disease were considered (i.e., smoking, age, and alcohol ingestion habits). The difference in the results may be from different experimental conditions, and further research is needed.

### Reproductive system

The influence of electromagnetic radiation on the reproductive system has been attracting scientists’ attention. Although numerous studies have been reported, only a few studies have focused on the SW band. Several studies found no significant association between congenital malformations of fetuses and SW radiation with non-thermal intensity [32, 33]. Oliveira et al. [33] conducted a study about the exposure of pregnant rats to SW electromagnetic athermal radiation. Five pregnant female rats were subjected to SW radiation with an average power of 4.5 W (similar to the exposure to clinical SW therapy) for 15 min daily and 20 days in total. After exposure, the fetuses were removed and detected. Compared with control fetuses, no cases of teratogenesis, stillbirth, or malformation of internal organs occurred in the exposure group. SW hyperthermic exposure may induce teratogenicity [34, 35]. Brown-Woodman et al. [34] exposed pregnant rats, at 9 days of gestation, to 27 MHz continuous SW radiation. The results showed that the exposed rats, with a temperature elevation of 5 °C in the rectum induced by radiation, had fetal malformation (microphthalmia, encephalocele, facial clefting and maxillary hypoplasia). Additionally, another epidemiological investigation revealed a significantly higher incidence of abnormal male reproduction symptoms in SW radiomen [36]. This study analyzed the reproductive health of 89 male soldiers with an average age of 22.9 who had worked in SW radiation working conditions for an average period of 3.2 years, and they had a relatively higher incidence of impotence, premature ejaculation and low sexual desire compared to the control group. This may indicate an adverse effect of long-term SW radiation on male reproductive health.

### Endocrine system

The endocrine system plays an important role in regulating various physiological functions, and numerous studies have focused on the effect of electromagnetic radiation on the incretion activity, especially the secretion of melatonin, gonadal hormone and adrenaline [24, 35, 37–40].

Melatonin is secreted by the pineal gland in mammals and human beings, and it has obvious circadian rhythmicity. Additionally, it plays a significant role in modulating sleep with higher blood levels during the night. An observational study found an association between decreased excretion of melatonin during the night and exposure to SW (6–22 MHz) electromagnetic radiation [24]. The nightly melatonin excretion in residents near a SW radio transmitter had a 10% decrease compared to baseline values and a 26% increase after the transmitter was shut down for 1 week along with an improved sleep quality in residents. This result was partly consistent with an earlier pilot study on dairy cattle at a farm near SW range (3–30 MHz) radio transmitter signals (the average exposed field intensity was 1.59 mA/m) [40]. Additionally, the secretion of gonadal hormone affects various physiological behaviors, such as estrus, mating, and gravidity, in mammals. A study found that there was a significant reduction in the number of rats that mated and the number of those that mated and became pregnant when these rats were exposed to 27.12 MHz SW radiation for 1 h/d for a total of 25 days [34]. This abnormal reduction probably resulted from disorder of gonadal hormone secretion, and SW radiation caused this dysfunction. Additionally, another study revealed a dose-dependent change in adrenaline secretion caused by SW radiation [40]. Rats were exposed to pulsed EMF at 27.12 MHz for 10 min/d and a total of 15 days. Then, structural and ultrastructural changes in the adrenal glands of rats were observed. When exposing rats at a 1/80 impluses/s radiation dose, a slight stimulation of adrenaline synthesis and secretion was found, which was beneficial. Additionally, radiation at a 4/400 impluses/s dose induced a higher secretion. However, a 6/600 impluses/s radiation significantly decreased the secretion with the appearance of lytic and cell necrotic foci.

In all, whether SW radiation is harmful for biological systems is closely related to the radiation dose or parameters. Nevertheless, prior studies have not reached an accordant conclusion on the dose-effect relationship between SW radiation and living organisms.

## Mechanisms of action

The interaction mechanisms of EMF with living organisms have been investigated for many years, and both experimental and theoretical data have been reported. Generally, thermal and non-thermal mechanisms have been widely accepted. Thermal mechanisms depend on the high energy delivered by electromagnetic waves. They have been the basis of pulsed SW diathermy, which has been in clinical use for decades. However, the non-thermal mechanisms interested us the most. It was well accepted that the cellular membrane was a primary target for electromagnetic radiation, and it contained abundant ion channels and receptors for signaling. Numerous cellular studies have focused on the effects of SW radiation on signal transduction pathways, and the calcium (Ca^2+^)-dependent signaling pathways have received the most attention [41–43]. Additionally, several studies have revealed some relationships between SW radiation and chromosome or cell cycle injury, suggesting there are other injury mechanisms [10, 36, 44].

### Ca^2+^-dependent signaling pathways

As a secondary messenger in cellular signal transduction, Ca^2+^ performs its functions by binding to calmodulin (CaM). Ca^2+^ binds to and activates CaM; then, the activated CaM combines with constitutive nitric oxide synthase (cNOS), inducing the activation of cNOS [41]. This series of processes catalyze the synthesis of nitric oxide (NO), which is an essential signaling molecule and exerts different kinds of biological functions directly or indirectly [45]. The direct function involves pro-inflammation responses by CaM/cNOS/NO signaling. The indirect functions depend on the activation of downstream target enzymes adenylyl cyclase (AC) and soluble guanylyl cyclase (sGC); then, activated AC and sGC catalyze the synthesis of cyclic adenosine monophosphate (cAMP) and cyclic guanosine monophosphate (cGMP), respectively, regulating multiple biological processes (i.e., inflammation, cell differentiation, apoptosis, blood flow, tissue repair, etc.) by CaM/nNOS/cAMP signaling and CaM/NO/cGMP signaling [41, 46, 47].

Some experimental studies of human articular chondrocytes have showed that, after exposure to pulse-modulated 27.12 MHz EMF for 30 min, the cells had significantly increased levels of NO, cGMP, and DNA. The changes could be blocked by antagonists of CaM and NOS [41, 48]. These results demonstrated the important role of CaM/NO/cGMP signaling in cell proliferation caused by SW-EMF. Another study demonstrated that the differentiation and survival of MN9D neuronal cells induced by pulse-modulated EMF could be mediated by CaM/nNOS/cAMP signaling [49]. In this study, a notable differentiation phenomenon, as well as the increased cAMP content, occurred in MN9D cells after 15 min of exposure to pulse-modulated EMF, and the NOS inhibitor blocked this effect. Furthermore, it was reported that the CaM/cNOS/NO signaling could downregulate interleukin (IL)-1β, inducing reduced inflammation and accelerating wound repair [3, 47]. The alteration of Ca^2+^-dependent signaling pathways might be from increased Ca^2+^ concentration or channels caused by EMF stimulation because of the voltage-dependent property of the calcium channel. However, Pilla et al. [41] held the view that the proposed mechanism resulted from an EMF effect on voltage-dependent Ca^2+^ binding to CaM. Namely, EMF promoted the binding of Ca^2+^ and CaM, which was the initiating agent of EMF effects.

### Cell cycle alteration

The cell cycle phase contains G_1_, S, G_2_ and M periods, and it involves a variety of biological processes, including DNA synthesis and chromosome division. Many chemical or physical factors, including EMF, could induce alterations of the cell cycle and harm the cells. Ding et al. [36] detected a significantly higher percentage of DNA strand breakage in sperm cells in SW radiomen. Similarly, mitotic spindle disruption in HCC cells receiving specific modulated 27.12 MHz EMF exposure was observed [10]. These results indicated an interruption of the cell cycle induced by SW radiation. Additionally, an early study exposed Chinese hamster ovary cells in 27 MHz continuous-wave EMF for 2 h to 5 or 25 W/kg. Then, the cytofluorometric assay revealed a time and dose dependent alteration in the cell cycle caused by EMF [44].

## Conclusions

This review presented the biological effects of SW in recent years. In all, the application of SW has been an effective treatment modality in numerous clinical areas. It has been used for treating cancer, wound, pain, etc. The treatment was noninvasive, and this kind of short-term, low-level exposure of SW had few adverse effects. Based on the present results and evidence, there were not many more hazards than benefits from SW exposure at levels below the recommended safety limits. Although studies about adverse effects were sparse, especially on the long-term effects of low-level SW exposure, there remains potential harm, and some epidemiology studies have indicated that SW radiation puts human health at risk. Compared with other electromagnetic waves, such as the microwave and power frequency, this high frequency SW radiation may induce special unknown biological effects. Therefore, more investigations are needed. The common goal of scientists has been to clarify these biological effects, expecting to achieve more benefits from SW with fewer risks. Further studies would help to achieve this goal.

## Abbreviations

AC: Adenylyl cyclase
CaM: Calmodulin
cAMP: Cyclic adenosine monophosphate
cGMP: Cyclic guanosine monophosphate
cNOS: Constitutive nitric oxide synthase
ECG: Electrocardiogram
EEG: Electroencephalogram
EMF: Electromagnetic field
HCC: Hepatocellular carcinoma
HF: High frequency
IL: Interleukin
NO: Nitric oxide
SAR: Specific absorption rate
sGC: Soluble guanylyl cyclase
SW: Shortwave
